# P-995. Increased Red Cell Distribution Width (RDW) is associated with culture positive sepsis and with increased need of escalation in respiratory support in very low birth weight infants more than seven days of life undergoing sepsis workup secondary to vitals instability in the form of multiple episodes of true apneas or bradycardia and desaturations associated with apnea

**DOI:** 10.1093/ofid/ofae631.1185

**Published:** 2025-01-29

**Authors:** Kedar Tilak, Aashka Patel

**Affiliations:** Children's Mercy Hospital, Kansas City, Missouri; Oklahoma Children's Hospital, Oklahoma City, Oklahoma

## Abstract

**Background:**

Apnea of prematurity is nearly universal among very low birth weight infants (VLBW). In VLBW infants, changes in clinical status usually occur due to sepsis or non-infectious causes and it is challenging to differentiate between the two. We as clinicians use irregularities in vital signs as an early warning sign of sepsis to facilitate early treatment and improve patient outcomes. Adults have used RDW as a marker of worsening respiratory status and mortality from acute respiratory distress syndrome (ARDS). In this study, we seek to investigate elevations in Red Cell Distribution Width (RDW) as markers for culture positive sepsis and need for escalation of respiratory support in VLBW neonates.

Association between RDW values and positive cultures

**Figure:**
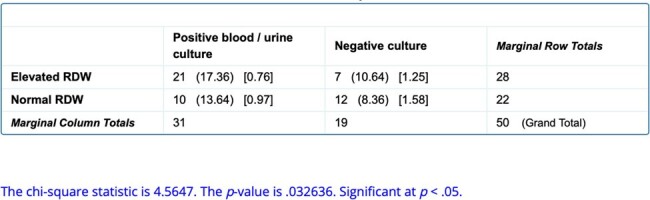

**Methods:**

We did a retrospective chart review using the Electronic Medical Records (EMR) of VLBW infants (< 1500g) admitted to the Neonatal Intensive Care Unit (NICU) of a community hospital over 3 years. We included all babies who had True apnea (apnea > 20 seconds) or apnea with bradycardia and desaturations, after day seven of life and had CBC tests sent and were being managed for presumed late onset sepsis secondary to this vital sign instability. Other demographic data was gathered and tabulated. The data was analyzed to check for association between increased red cell distribution width (RDW) and need for escalation of respiratory support. Escalation in respiratory support included an increased need of assisted ventilation to support breathing. We also looked at positive blood / urine cultures and RDW values.

Association between RDW values and change in baseline respiratory support

**Figure:**
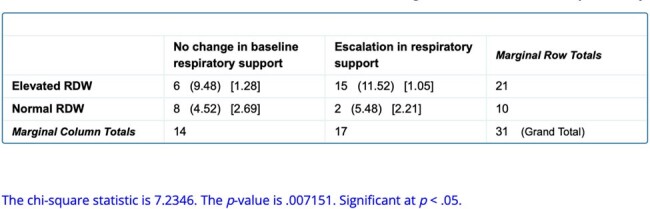

**Results:**

50 babies matched our inclusion criteria. 31 babies had a positive blood /urine culture. 21 of the 31 had elevated RDW which was statistically significant at p< 0.05 (Table1), hence showing that positive culture late onset sepsis had elevated RDW. Also, we saw increased need for escalation in respiratory support amongst culture positive babies with an elevated RDW at p < 0.05 (Table 2).

**Conclusion:**

We conclude from this, that elevations in RDW is a marker for VLBW babies needing escalation in respiratory support. Elevations were also seen in culture positive sepsis. More studies need to be done as most of the available data is in adults. We can look more associations in babies with RDS (respiratory distress syndrome) and predict respiratory distress and for early interventions and better outcomes.

**Disclosures:**

**All Authors**: No reported disclosures

